# Percutaneous Left Atrial Appendage Closure: Supporting Evidence, Limitations and Future Directions

**DOI:** 10.3390/jcm14072300

**Published:** 2025-03-27

**Authors:** Giuseppe Imperatore, Stijn Lochy, Mohamed Ben Yedder, Roberto Galea, Adel Aminian

**Affiliations:** 1 Health Science Interdisciplinary Centre, Sant’Anna School of Advanced Studies, 56127 Pisa, Italy; giuseppe.imperatore@santannapisa.it; 2 Department of Cardiology, Universitair Ziekenhuis Brussel (UZ Brussel), 1090 Brussels, Belgium; stijn.lochy@uzbrussel.be; 3 Department of Cardiology, Centre Hospitalier Universitaire de Charleroi, 6042 Charleroi, Belgium; mohamed.benyedder@humani.be; 4 Department of Cardiology, Bern University Hospital, Inselspital, University of Bern, 3012 Bern, Switzerland; roberto.galea@insel.ch

**Keywords:** atrial fibrillation, left atrial appendage closure, device, stroke prevention

## Abstract

Percutaneous Left Atrial Appendage Occlusion (LAAO) has emerged as a promising intervention for stroke prevention in patients with atrial fibrillation who are contraindicated for long-term anticoagulation therapy. Despite its growing adoption, a comprehensive review of the LAAO procedure is essential to consolidate the supporting evidence, identify limitations, and outline future directions. This review aims to evaluate the efficacy and safety of LAAO, drawing on clinical trials and real-world studies to provide a balanced perspective. Additionally, we address the limitations of current research, including variability in patient selection, procedural techniques, and follow-up protocols. By highlighting gaps in the knowledge and areas for improvement, this review aims to guide future research efforts to optimize and expand the therapeutic potential of LAAO.

## 1. Introduction

In Europe, 1–3% of the population currently suffers from atrial fibrillation (AF) [1]. With the progressively ageing population, AF is rapidly becoming a disease with epidemic proportions and is particularly feared for its thromboembolic potential, as 20% to 30% of all ischemic strokes are caused by AF [1,2]. Since their first approval in 2010, Direct Oral Anticoagulants (DOAC) have emerged as the leading pharmacotherapy for stroke prevention in AF patients [1]. DOACs provide both clinicians and patients with an effective and more convenient treatment option compared to Vitamin K Antagonists (VKA), the previous standard of care in anticoagulation [2,3]. Nevertheless, even with DOACs, major bleeding events remain a clinically relevant problem, with a yearly rate of 2 to 3.5%.

Percutaneous Left Atrial Appendage Occlusion (LAAO) is increasingly used as an alternative to oral anticoagulation (OAC) in patients with AF, particularly those with contraindications to these therapies [4], with its use growing exponentially worldwide [5,6]. Patient selection remains crucial, and ongoing clinical trials will provide evidence of its use in extended patient categories. Of note, several ongoing trials will be evaluating LAAO as a direct alternative to oral anticoagulants in patients at low bleeding risk [7,8]. To this end, procedural safety and efficacy must be maximized through device improvements and enhanced preprocedural, intraprocedural, and follow-up imaging, which are essential for maintaining the immediate and long-term efficacy of LAAO intervention [4,9]. In this review, we will summarize the supporting evidence, remaining limitations, and future directions in the field of transcatheter LAAO [4,9].

## 2. Supporting Evidence

While OAC remains the standard of care for stroke prevention in AF patients, there is a growing body of evidence supporting LAAO as an effective alternative or complementary treatment.

To date, only three moderately sized RCTs, comparing LAAO with OAC therapy, have been published and have supported the effectiveness and overall safety of LAAO, particularly when compared with VKA [10]. The meta-analysis of the PROTECT AF and PREVAIL trials demonstrated that LAAO with the first iteration of the Watchman device was non-inferior to warfarin for the combined primary efficacy endpoint of stroke, systemic embolism, and cardiovascular death [10,11,12]. Notably, there was a reduction in fatal and severe strokes post-LAAO, although it was based on small event numbers. Cardiovascular and overall mortality were lower with LAAO compared to warfarin. Safety analysis showed similar overall major bleeding rates, but significantly fewer non-procedure-related major bleeding events.

Overall, the meta-analysis has provided enough evidence to support the Watchman device as an effective alternative to long-term warfarin therapy for stroke prevention and has led to the 2015 FDA approval of this device in patients that “have an appropriate rationale to seek a nonpharmacological alternative to warfarin” [7,12].

The multicentre, prospective, randomized PRAGUE-17 trial is currently the only published trial comparing the efficacy and safety of LAAO versus DOAC in high-risk AF patients. After a median follow-up of 3.5 years, the composite primary endpoint of stroke/TIA, systemic embolism, clinically significant bleeding (the International Society on Thrombosis and Haemostasis definition), and cardiovascular mortality was observed in 8.6% in the LAAC group and 11.9% in the NOAC group (*p* = 0.006 for non-inferiority). No differences were determined in any of the components of the primary endpoint. Although the study did not find a difference in overall bleeding events, severe non-procedural bleeding events were significantly reduced in the LAAO group (3.4% vs. 5.9%; *p* = 0.039).

A meta-analysis of the three currently available RCTs comparing LAAO vs. OACs (PROTECT-AF, PREVAIL, and PRAGUE-17), showed no significant difference in stroke or thromboembolic risk between the two strategies. However, LAAO was associated with a lower risk of haemorrhagic strokes, cardiovascular mortality, overall mortality, and fatal stroke. Additionally, evidence from large multicentre registries such as EWOLUTION and AMULET prospective global observational study supports the overall safety and efficacy of LAAO in high-risk AF populations, showing a significant reduction in stroke and bleeding events, as compared to expected rates based on CHA2DS2-VASc and HASBLED Scores.

With regard to device comparisons, the AMULET IDE trial demonstrated the non-inferiority of the Amulet device compared to the Watchman 2.5 device in preventing stroke and systemic embolism, leading the FDA to approve the use of the Amulet device in the US. In the smaller-sized multicentre SWISS APERO trial, the Amulet device was associated with lower rates of Peridevice Leaks (PDL) at 45 days transoesophageal echocardiography (TEE) follow-up, but higher procedure-related complications rates, and similar clinical outcomes at 45 days compared with Watchman 2.5/Flx. 

Several ongoing randomized clinical trials are comparing LAAO with the best medical care, including DOACs, in various clinical settings (Table 1).

Despite these advancements, there are still open questions and limitations regarding the optimal therapeutic approach, and the management of post-procedural complications such as device-related thrombus (DRT) or significant PDL.

Overall, the currently available evidence underscores the evolving landscape of LAAO, with ongoing research aimed at optimizing patient outcomes and refining device technologies.

## 3. Procedural Optimization: What Are the Main Determinants?

### 3.1. Preprocedural Planning

Appropriate planning and guidance for LAAO are crucial to a successful procedure. While TEE is still widely used for preprocedural planning, Cardiac Computed Tomography (CT) and its newer platforms are emerging as an increasingly used imaging alternative.

Since the anatomy of the LAA is highly variable, detailed spatial resolution and three-dimensional (3D) assessment are essential to minimize the risk of device missizing and anticipate complex anatomies. While the largest LAA diameters are typically used for device sizing, and are still recommended by device manufacturers, the use of mean LAA diameters could represent a better alternative in case of a highly eccentric LAA landing zone.

Traditionally, 2D-TEE-guided device size selection has followed uniform oversizing [13], by scanning the LAA from 0° to 135°, but a pure 2D approach will tend to underestimate the LAA orifice area compared to 3D-imaging modalities, and both 2D and 3D TEE methods exhibit significant inter-operator variability [14]. Due to its better spatial resolution, CT allows not only for a precise assessment of the LAA orifice and landing zone dimensions but also for the LAA relationship with important surrounding structures [15,16]. Improvement in sizing accuracy is essential to optimize procedure efficiency by reducing procedural time, radiation exposure, the number of devices used, and the total contrast volume.

In summary, any 3D imaging modality (CT or 3D-TEE) should be preferred over 2D-TEE and mean diameters should be strongly considered for device oversizing in the case of a highly eccentric landing zone. When available, CT can provide the most reliable LAA diameters and could become the recommended imaging modality for guiding device selection in the future [17].

The future of preprocedural planning imaging for LAAO is promising, with advancements in imaging techniques expected to enhance procedural success and patient outcomes. Future techniques, such as computational modelling and CT/fluoroscopy fusion imaging, will refine device selection, sizing, and positioning [18]. Additionally, preprocedural imaging with CT has been associated with improved procedural success rates, although its impact on major adverse events remains to be proven [18,19,20,21,22,23].

With regard to the added value of CT-based artificial intelligence for LAAO preplanning, the recently published PREDICT-LAA trial focused on comparing the impact of FEops HEARTguide™, a CT-based simulation planning tool, on the procedural efficiency and clinical outcomes of LAAO procedures, as compared to “standard of care” preprocedural CT assessment [24]. Importantly, the use of FEops resulted in a two-fold increase in procedural success with a single device deployment, and with a 25% reduction in the use of radiation and contrast medium, demonstrating improved procedural efficiency. The use of FEops preplanning was also associated with a 40% increase in complete LAA seal and an 80% reduction in the risk of DRT [24,25].

### 3.2. Procedural Imaging: Moving Towards a Mini-Invasive Approach Without General Anesthesia?

Intracardiac echocardiography (ICE) imaging is an emerging alternative to TEE for LAAO guidance. It allows the use of local anesthesia, avoiding intubation and its complications, improving cath lab workflow, enabling faster recovery, and facilitating early discharge [26,27]. Multiple studies on ICE-guidance for LAAO have demonstrated excellent procedural success and effectiveness rates, with minimal overall complications, except for a small but significant increase in the risk of pericardial effusion as compared to TEE-guidance, with this risk being directly proportional to operator’s experience [22]. Despite these advantages, ICE-guided LAAO remains underutilized due to inherent limitations, which mainly include the cost of the probes (which is non-reusable) but also the non-negligible learning curve. Future research is needed to assess if these remaining drawbacks could be mitigated by newer 4D and softer ICE catheters [6,22].

Like ICE guidance, the use of miniaturized TEE probes (micro and mini TEE) allows for LAAO guidance under local anesthesia, and has been shown to be a safe and effective strategy, demonstrating a high technical success rate and low complication rates [28,29,30]. These miniaturized TEE probes have the potential to provide adequate image quality to visualize LAA anatomy and important surrounding structures, but with a faster learning curve than ICE, reducing thereby procedural time and costs, with the same potential of improving cath lab workflow [28]. Most likely, these miniaturized imaging approaches will gradually replace standard TEE guidance [29,30]. The recent introduction of 3D mini-TEE is a further step in the expansion of this approach by allowing detailed visualization of the LAA from multiple cross-sectional planes, which is crucial for accurate sizing and device positioning [31]. Similarly, the advent of 4D-ICE combines the advantages of 2D-ICE guidance with better spatial resolution, with the potential to lower complication rates and give more direct feedback to the operator [32]. A recent meta-analysis [30] compared ICE and TEE for LAAO and found that ICE-guided procedures had higher technical success rates and required fewer devices. However, ICE was associated with higher rates of adverse events, such as pericardial effusion and vascular complications. While ICE shows promise for higher success and efficiency, the increased risk of complications suggests the need for careful patient selection and further research to fully understand its long-term safety and outcomes. Altogether, by improving procedural workflow, these new minimally invasive imaging approaches will be useful to fulfil the need to perform a greater number of procedures in the future, while maintaining efficiency and safety.

### 3.3. Procedural Volume and Its Importance in LAAO Outcomes

The procedural volume (the total number of procedures performed within a medical centre) and annual volume per operator (the number of procedures performed by an operator within a year) are critical factors that influence the success rates, safety, and outcomes of LAAO procedures. It is well-accepted that high-volume centres and operators tend to have lower rates of complications, a higher likelihood of successful implantation, and faster recovery times [33]. However, it is essential to address challenges related to access, training, and quality assurance to ensure that all patients receive the same highest standard of care [34].

## 4. Device-Related Thrombus: From an Innocent Bystander to a Clinical Dilemma

The diagnosis of Device-Related Thrombus (DRT) relates to thrombus formation on the atrial surface of the LAAO device after implantation. Although the process of DRT formation remains not completely understood, several anatomical, device, and hemodynamic factors have been suggested to increase thrombogenicity [34].

The incidence of DRT changes among studies because of the variability in the frequency and standardization of post-LAAO imaging [35]. Several large studies have defined an incidence in the range of 2 to 5% [35]. Specifically, the rate of stroke or systemic embolism in these patients is approximately 3–5 times higher than in those without DRT [36].

Also, DRT has been subcategorized into Early DRT (detection within 6 months after LAAO) and Late DRT (later than 6 months) [37]. European guidelines recommend imaging FU at 6–24 weeks post-procedure [34,37]. Early detection and management are crucial to mitigate the risk of DRT-related adverse events.

### 4.1. Which Patients Are at Higher Risk of Developing DRT?

Identifying risk factors for DRT on an individual basis remains challenging. Prior stroke or TIA and permanent AF are the most consistent “patient-related” DRT predictors, while implantation depth is the most consistent “procedural” risk factor. A novel approach for DRT prediction harnesses computational flow dynamics to identify patients at risk. Based on the hypothesis that thrombus formation on LAAO devices is flow-related [38], this approach might provide patient-specific recommendations on the optimal device selection to minimize DRT risk [35,36,37,38]. An overview of potential predictors is depicted in Table 2. Furthermore, Mesnier et al. [39] provided definitions of “resolved” DRT, documented by at least one imaging examination; “persistent” DRT, as the continued presence of DRT in all imaging examinations; and “recurrent” DRT, as a new onset DRT after at least one imaging study documented resolution of the initial DRT. The combination of persistence or recurrence is considered as an unfavourable evolution of DRT. In the LAAO-DRT multicentre registry, one-third of DRT had an unfavourable evolution and this was associated with a two-fold increased risk of TE events, with a larger initial DRT (≥7 mm) size being an independent predictor of persistent/recurrent DRT [39,40].

### 4.2. A Modifiable Risk Factor: Device Implantation Depth

Deep device implantation is defined as an incomplete coverage of the pulmonary vein ridge, leaving an uncovered rim of at least 10 mm on TEE or CT images [41,47]. In a large multicentre registry, LAAO device implantation depth has proven to be an independent risk factor for DRT, regardless of device type (with or without proximal disc), with deeper device implantation and larger uncovered LAA areas being associated with the highest incidence of DRT [46]. Regarding the physiopathological link, it is assumed that deep device implantation leaves residual appendage with blood swirling and stasis, especially in the untrabeculated area between the upper edge of the device and the pulmonary ridge. Given its clinical impact, avoiding deep device implantation could represent an important and future “DRT-preventive” procedural target in patients undergoing LAAO [46].

### 4.3. DRT Detection: Are TEE and CT Equivalent?

**Figure 1:**
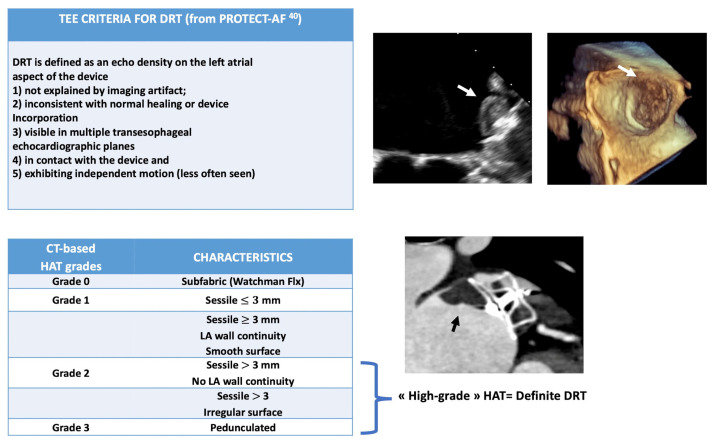
DRT Criteria for TEE and CT imaging modalities. TEE = Transesophageal Echocardiography; CT = Computed Tomography; HAT = Hypoattenuated Thickening; LA = Left Atrial; DRT = Device-Related Thrombus.

Although TEE is still considered as the gold standard for the diagnosis of DRT, CT is increasingly used for post-procedural imaging follow-up, including DRT detection [48,49]. However, the higher resolution of CT in post-LAAO surveillance has given rise to findings with sometimes unclear clinical implications, such as Hypoattenuated Thickening (HAT). A key challenge in DRT diagnosis by CT stands from the lack of a standard methodology to categorize HAT. However, Korsholm et al. [18,49] recently proposed an international consensus aiming at standardizing the classification of CT-detected HAT based on size, morphology, and location of findings, as well as the specific device design. The identification and characterization of DRT on CT is based on the observation of “high-grade” HAT on the atrial device surface [34,49]. A comparison between TEE and CT criteria for the diagnosis of DRT is depicted in Figure 1.

### 4.4. The Controversial Aspects of Antithrombotic Therapy for DRT Prevention

The association of post-LAAO antithrombotic strategy and DRT remains an open and challenging question. Antithrombotic therapy prevents DRT during the device healing process, as DRT might result from incomplete prosthesis endothelialisation. Achieving an optimal balance between thrombotic and bleeding risks related to antithrombotic therapy is crucial to identifying the best strategy for the wide spectrum of patients undergoing LAAO.

Several observational studies have reported that a single APT after LAAO may be safe, without an increased risk of DRT or stroke [50]. Given the scarce evidence available, this approach should be only used in selected patients who may not tolerate DAPT or DOAC, carefully weighing the risks of DRT versus major bleedings [38,50].

DOAC may be a valid alternative for patients at low or intermediate bleeding risk. However, current evidence regarding the efficacy and safety of DOAC remains limited and primarily comes from non-randomized studies. While the optimal dosing regimen for DOAC is still debated, current evidence suggests that half-dose DOAC regimens might represent an optimal strategy for selected patients [51,52]. Notably, short-term use of DOAC has been shown to significantly reduce coagulation activation compared to DAPT, an important concept for managing DRT risk.

The recently published ADALA study aimed to compare low-dose DOAC with DAPT after LAAO [52]. After enrolling 90 patients, the study found that low-dose DOAC was associated with fewer DRT and major bleeding events compared to DAPT. However, the small sample size and the premature termination of the study due to low enrollment limit the reliability of these findings [18,53]. Overall, current evidence with DOACs after LAAO is promising but still inconclusive, highlighting the need for larger trials to validate the efficacy and safety of DOACs in this context [53].

It is important to remember that the high bleeding risk of most patients undergoing LAAO still represents a limiting factor for any intensive long-term antithrombotic therapy. Any future “gold standard” strategy will need to provide a consistent balance between efficacy (DRT prevention) and safety (bleeding prevention) [52,53].

## 5. Peridevice Leaks (PDL): From Detection to Recent Insights into Clinical Implications

Peridevice Leak is defined as a residual communication between the left atrium and the LAA [54]. Traditionally, PDL visualization has relied on TEE, where the severity is determined by the width of the colour Doppler jet, with thresholds for clinical significance arbitrarily set at either 3 or 5 mm [34,54]. With regard to imaging modality, studies have demonstrated that CT is a more sensitive method for detecting and discerning the mechanism of post-LAAO leaks. Currently, CT-based residual LAA patency indicates increased contrast medium density distal to the LAAO device [54].

Of note, CT tends to report larger PDL sizes compared to TEE, though there is no clear correlation between the two imaging modalities [34,54]. Moreover, despite its higher sensitivity, the definition of a “significant” CT-based PDL size remains unclear, whereas TEE considers a width of 3 to 5 mm as “large” or “significant” [24,54]. A comparison of TEE and CT for post-LAAO leaks is depicted in Table 3. Whatever the imaging modality used, the clinical significance of post-LAAO residual leaks is an ongoing topic of debate. Recent analyses from the NCDR LAAO registry [54,55] suggest that small TEE leaks (<5 mm) may be clinically relevant. Conversely, Samaras et al. have shown in a large meta-analysis that all PDL detected by TEE are actually associated with an increased risk of thromboembolism, with a positive graded association between PDL size and risk across various TEE cut-offs. In this large meta-analysis, although larger leaks (≥5 mm) were associated with the highest risk, even smaller leaks (≥3 mm) significantly increased the risk of thromboembolism [42,43,56,57]. Altogether, the therapeutic decision pathway is complicated by the lack of standardization in diagnosis and imaging [44,45], and by the lack of consensus on which leaks are clinically significant, and by the risk of bleeding associated with prolonged antithrombotic therapy in case of incomplete sealing [58,59,60]. Current interventional approaches to close residual PDL include vascular plugs, detachable coils, and radiofrequency ablation, but the selection of which PDL requires closure and the optimal closure technique is not well defined [61,62,63,64].

## 6. Conclusions

Over the last two decades, percutaneous LAAO has demonstrated substantial efficacy in reducing thromboembolic risks for AF patients who are unsuitable for long-term OAC.

Evidence from RCTs and large registries indicates that LAAO is non-inferior to OAC in preventing stroke and systemic embolism, with additional benefits in reducing all-cause mortality. However, the procedure is not without limitations. Procedural and post-procedural complications, although decreasing over time, still pose significant risks owing to the exponential growth of these procedures worldwide. The optimal post-procedural antithrombotic regimen remains an area of ongoing research. Additionally, the cost-effectiveness of LAAO compared to lifelong oral anticoagulation is still debated, especially with the impending expiration of DOAC patents.

By enabling precise device selection and positioning, advanced imaging techniques, including 3D modalities and artificial intelligence-based computational modelling, will become essential for pre-procedural planning, procedure guidance and post-procedural surveillance, reducing thereby the risk of complications and enhancing overall safety and efficacy of LAAO procedures.

Future research should focus on refining patient selection to maximize the benefits of LAAO, exploring new device technologies and establishing standardized post-procedural care protocols. Long-term studies are needed to assess the durability and effectiveness of LAAO devices over time.

## Figures and Tables

**Table 1:** Overview of ongoing major trials comparing LAAO and medical management.

Trial	Patients (Randomisation)	Main Inclusion Criteria	Primary Endpoint
Patients with high bleeding risk, LAAO vs. best medical care			
CLOSURE AF (NCT028301521)	1000 (1:1)	Non-valvular AF (CHA2DS2VASc ≥ 2) and high risk for bleeding: HAS-BLED ≥ 3 S/P bleeding BARC ≥ 3 Recurrent bleeding as contraindication for long-term (N)OAC Chronic renal insufficiency (eGFR 15–29 mL/min/1.73 m^2^)	Survival free of events (combined endpoint: stroke, SE, bleeding, CV or unexplained death > 2 years)
COMPARE LAAO (NCT04676880)	609 (2:1)	Non-valvular AF (CHA2DS2-VASc score ≥ 2), non-eligible for long-term OAC	Time to first occurrence of stroke; Time to combined endpoint: stroke, TIA, or SE; Peri-procedural complications
Patients without high bleeding risk, LAAO vs. DOAC			
CHAMPION AF (NCT04394546)	3000 (1:1)	Non-valvular AF (CHA2DS2VASc score ≥ 2), eligible for long-term OAC	Non-inferiority for combined endpoint: stroke, CV death < 36 months; superiority non-procedural bleeding < 36 months; non-inferiority for combined endpoint: ischaemic stroke, SE < 60 months
CATALYST (NCT04226547)	2650 (1:1)	Non-valvular AF (CHA2DS2-VASc score ≥ 3), eligible for long-term OAC	Non-inferiority of combined endpoint: ischaemic stroke, SE and CV mortality at 2 years; superiority: clinically relevant bleeding, excluding procedure-related bleeding at 2 years; non-inferiority of combined endpoint: ischaemic stroke, SE at 3 years
Post-intracerebral bleeding status, LAAO vs. best medical care			
CLEARANCE (NCT05063409)	500 (1:1)	Non-valvular AF (CHA2DS2VASc score ≥ 2), status post-intracerebral bleeding > 6 weeks	Combined endpoint: stroke, SE, bleeding or CV/unexplained death (2-year FU)
STROKECLOSE (NCT02830152)	750 (2:1)	Non-valvular AF (CHA2DS2VASc score ≥ 2), status post-intracerebral bleeding > 4 weeks but <6 months before randomization	Combined endpoint: stroke, SE, bleeding or death (5-year FU)
Post-ischaemic stroke/TIA status, LAAO vs. DOAC			
OCCLUSION AF (NCT03642509)	750 (1:1)	Non-valvular AF (CHA2DS2VASc score ≥ 2), eligible for long-term OAC. S/P stroke/TIA within 6 months before randomization	Combined endpoint: stroke, SE, bleeding, or death (5-year FU)
ELAPSE (NCT05976685)	482 (1:1)	Recent (≤3 months) symptomatic stroke., active and ongoing OAC therapy at stroke onset not stopped/paused for >48 h due to any reason, Active or planned long-term therapy with DOAC	Composite of recurrent ischemic stroke, systemic embolism, or cardiovascular death (whatever comes first).
LAAO and terminal renal insufficiency			
LAA-Kidney (NCT05204212)	430 (1:1)	Non-valvular AF (CHA2DS2VASc ≥ 2), end-stage chronic kidney disease (GFR < 15 mL/min/1.73 m^2^)	Combined endpoint of stroke, SE, CV/unexplained death and major bleeding (≥3 BARC) through 18 FU months
LAAO plus OAC vs. OAC alone			
LAAOS-4 (NCT059636989)	4000 (1:1)	AF in patients with a history of ischemic stroke or SE CHA2DS2-VASc ≥ 4. OAC therapy for at least 3 months	Ischemic stroke/SE

Abbreviations: AF = atrial fibrillation; DOAC = Direct Oral Anticoagulant; SE = systemic embolism; TIA = Transient Ischemic Attack; FU = follow-up; BARC = Bleeding Academic Research Consortium score; CV = cardiovascular.

**Table 2:** Overview of predictors of DRT formation after LAAO.

First Author (Year)	Study Design (Patients)	Patient-Related Predictors	Procedural/Post-Procedural Predictors
Kaneko et al. [41] (2017)	Single-centre analysis (78)	CHA_2_DS_2_-VASc score *	device implantation depth *
Dukkipati et al. [36] (2018)	Ad hoc analysis of PROTECT-AF and PREVAIL trial (1739)	LAA orifice width *, permanent AF *, prior stroke/TIA *, LV dysfunction *, vascular disease *	
Pracon et al. [41] (2018)	Single-centre analysis (99)	LV dysfunction, previous VTE	Device size, device implantation depth
Fauchier et al. [37] (2018)	Multicentre registry (France) (469)	Older age *, prior stroke/TIA *	No DAPT or OAC at discharge *
Aminian et al. [42] (2019)	Prospective global Amulet registry (1088)	LAA orifice width *	
Simard et al. [43] (2021)	Global DRT registry (711)	Hypercoagulopathy *, permanent AF *, renal insufficiency *	Pericardial effusion *, device implantation depth *
Schmidt et al. [44] (2022)	Ad hoc analysis of Amulet IDE trial (1788)	Older age *, female sex *, AF the time of the procedure *	
Vij et al. [45] (2022)	Multicentre registry EUROC-DRT (537)	Older age *, prior stroke/TIA *, SEC *	
Freixa et al. [46] (2023)	Multicentre registry (n1317)		Device implantation depth *, no or single APT post-LAAO *

* Indicates independent predictors on multivariate analysis. Abbreviations: LAAO = Left Atrial Appendage Occlusion; VTE = Venous Thromboembolism; TIA = Transient Ischemic Attack; DAPT = Dual Antiplatelet Therapy; OAC = Oral Anticoagulant; DRT = Device-Related Thrombus; SEC = Spontaneous Echo Contrast; LV = Left Ventricular.

**Table 3:** Comparison of TEE and CT for post-LAAO leaks.

	TEE	CT
DEFINITION/METHODS	◾ Detection of residual blood flow around the occlusion device ◾ Use colour Doppler (Nyquist scale between 20 and 50 cm/s) ◾ The width of jet is measured in multiple views ◾ The largest measurement defines severity	◾ Presence of contrast distal to the LAAO device = Residual LAA patency ◾ Presence of LAA density ≥ 100 HU and/or LAA density ≥ 25% of that of the LA
	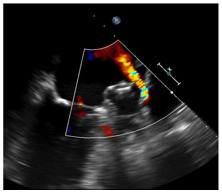	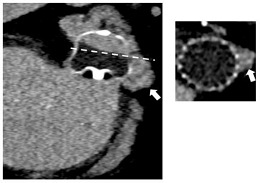
LIMITATIONS	◾ Interobserver variability ◾ Unclear cutoff to define severity of PDL: 3 mm? 5 mm?	◾ Highly sensitive and can detect even small leaks missed by other imaging modalities ◾ Discrimination between “intra-device” LAA Patency (lack of endothelialization vs. microgaps) and PDL ◾ Cutoff for severity not well defined ◾ Unclear prognostic impact
INCIDENCE *	PDL: 26% [24]	LAA Patency: 55% [24] PDL: 57% [24]

Abbreviations: TEE = Transeophageal Echocardiography; CT = Computed Tomography; PDL = PeriDevice Leak; LAA = Left Atrial Appendage. * Derived from Samaras et al. meta-analysis.

## Abbreviations and Acronyms

LAA	Left Atrial Appendage
DRT	Device-Related Thrombus
PDL	Peri-Device Leak
LUPV	Left Upper Pulmonary Vein
PR	Pulmonary Ridge
TEE	Transesophageal Echocardiography
CT	Computed Tomography
HAT	Hypo-attenuated Thickening
AF	Atrial Fibrillation
SE	Systemic Embolization
TIA	Transient Ischemic Attack
